# Optimized HepaRG is a suitable cell source to generate the human liver chimeric mouse model for the chronic hepatitis B virus infection

**DOI:** 10.1038/s41426-018-0143-9

**Published:** 2018-08-10

**Authors:** Lunzhi Yuan, Xuan Liu, Liang Zhang, Yali Zhang, Yao Chen, Xiaoling Li, Kun Wu, Jiali Cao, Wangheng Hou, Yuqiong Que, Jun Zhang, Hua Zhu, Quan Yuan, Qiyi Tang, Tong Cheng, Ningshao Xia

**Affiliations:** 1 State Key Laboratory of Molecular Vaccinology and Molecular Diagnostics, National Institute of Diagnostics and Vaccine Development in Infectious Diseases, School of Life Science, School of Public Health, Xiamen University, 361102 Xiamen, P. R. China; 2 Department of Microbiology, Biochemistry and Molecular Genetics, New Jersey Medical School, Rutgers University, 225 Warren Street, Newark, NJ 070101 USA; 3 Department of Microbiology, Howard University College of Medicine, Washington, DC 20059 USA

## Abstract

The human liver chimeric mouse with primary human hepatocytes (PHHs) engraftment has been demonstrated to be a useful animal model to study hepatitis B virus (HBV) pathogenesis and evaluate anti-HBV drugs. However, the disadvantages of using PHHs include the inability for cellular expansion in vitro, limited donor availability, individual differences, and ethical issues, necessitating the development of alternatives. To obtain in vitro expandable hepatocytes, we optimized the hepatic differentiation procedure of the human liver progenitor cell line, HepaRG, using four functional small molecules (4SM) and enriched the precursor hepatocyte-like cells (HLCs). HepaRG cells of different hepatic differentiation states were engrafted to immunodeficient mice (FRGS) with weekly 4SM treatment. The HepaRG-engrafted mice were challenged with HBV and/or treated with several antivirals to evaluate their effects. We demonstrated that the 4SM treatment enhanced hepatic differentiation and promoted cell proliferation capacity both in vitro and in vivo. Mice engrafted with enriched HepaRG of prehepatic differentiation and treated with 4SM displayed approximately 10% liver chimerism at week 8 after engraftment and were maintained at this level for another 16 weeks. Therefore, we developed a HepaRG-based human liver chimeric mouse model: HepaRG-FRGS. Our experimental results showed that the liver chimerism of the mice was adequate to support chronic HBV infection for 24 weeks and to evaluate antivirals. We also demonstrated that HBV infection in HepaRG cells was dependent on their hepatic differentiation state and liver chimerism in vivo. Overall, HepaRG-FRGS mice provide a novel human liver chimeric mouse model to study chronic HBV infection and evaluate anti-HBV drugs.

## Introduction

Hepatitis B virus (HBV) is an important globally spreading pathogen and infects >350 million people worldwide. Although prophylactic vaccine and drug regimens to suppress viremia are available, chronic HBV infection can rarely be cured^1–3^. HBV has an extremely narrow host range and hepatic tropism, and it only productively infects human and a few primates’ hepatocytes^4–6^. Thus, a small animal model for HBV is difficult to set up, although it is critical for studying HBV biology and the development of novel antivirals. Currently used animal models for HBV infection are the human liver chimeric mice generated by engrafting primary human hepatocytes (PHHs) or hepatocyte-like cells (HLCs) to the livers of immunodeficient mice^7–14^. However, PHH proliferates very slowly, and it is difficult to maintain its differentiated hepatic state in vitro. In addition, PHHs from different individuals often cause varied scales of liver chimerism and outcomes of HBV infection in PHH-engrafted mice^15–19^. Therefore, an in vitro expandable and hepatic differentiated cell line that is permissive for HBV infection is the ideal alternative for PHHs to generate a better human liver chimeric mouse.

The bipotent human hepatic progenitor cell line HepaRG can differentiate into either HLCs or cholangiocyte-like cells (CLCs) and has been widely used for HBV infection for more than a decade^20,21^. To fully support HBV infection and replication, HepaRG cells were subjected a classical 4-week hepatic differentiation procedure using dimethyl sulfoxide (DMSO). The HepaRG-derived HLCs were demonstrated to be permissive for HBV infection in vitro, whereas the CLCs were not^22^. Therefore, HepaRG-derived HLCs have been widely accepted as a cell model for antiviral drug development and evaluation^23–25^. Indeed, HepaRG cells were engrafted to mouse liver, but the chimerism of the liver reconstituted with HepaRG cells was extremely low due to the poor proliferation in vivo^26^. The capacity of HepaRG cells to support HBV infection in vivo remains unknown. Previous studies have demonstrated that a certain ratio of liver chimerism and hepatic differentiation are important to support chronic HBV infection in human liver chimeric mice;^16,27^ hence, an enhancement of hepatic differentiation and cell proliferation is required to establish the HepaRG-engrafted mice.

Recently, several small molecules have demonstrated outstanding effects on hepatic differentiation and cell proliferation. First, FPH1 and FPH2 were found to induce proliferation of PHHs in vitro^28^. Second, FH1 was able to enhance hepatic differentiation of stem cells^28^. Furthermore, XMU-MP-1 augmented PHH proliferation by targeting kinases MST1 and MST2 and activating hippo signaling in vivo^29^. Moreover, collagenase IV has been shown to enrich the hepatocyte marker human albumin (hALB) and α-1-antitrypsin (hAAT) double-positive (DP) cells during the generation of HLCs by direct programming and to generate a high ratio of precursor HLCs with relatively mature hepatic differentiation^30^. Despite their striking effect, the four small molecules (4SM), FPH1, FPH2, FH1 and XMU-MP-1, as well as the cell enrichment protocol have not yet been applied in hepatic differentiation procedures for HepaRG cells or the generation of human liver chimeric mice.

Here we optimized an in vitro hepatic differentiation procedure for HepaRG cells by 4SM treatment and enrichment of DP cells. HepaRG cells in different hepatic differentiation states were characterized in vitro and engrafted to immunodeficient *Fah*^*-/-*^*Rag2*^*-/-*^*IL-2Rγc*^*-/-*^ SCID (FRGS) mice. The 4SM treatment enhanced both the hepatic differentiation and proliferation capacity of HepaRG cells in vitro and in vivo, which is required for HBV infection. FRGS mice engrafted with DP prehepatic differentiated HepaRG cells and 4SM treatment, named HepaRG-FRGS mice, were adequate to support HBV chronic HBV infection with detectable HBV components, including HBV surface antigen (HBsAg), e antigen (HBeAg), core antigen (HBcAg), RNA, DNA and covalently closed circular DNA (cccDNA). The HepaRG-FRGS mice were also used to evaluate antivirals, such as novel host target agents (HTAs).

## Results

### Optimization of the hepatic differentiation of HepaRG cells with 4SM

**Fig. 1:**
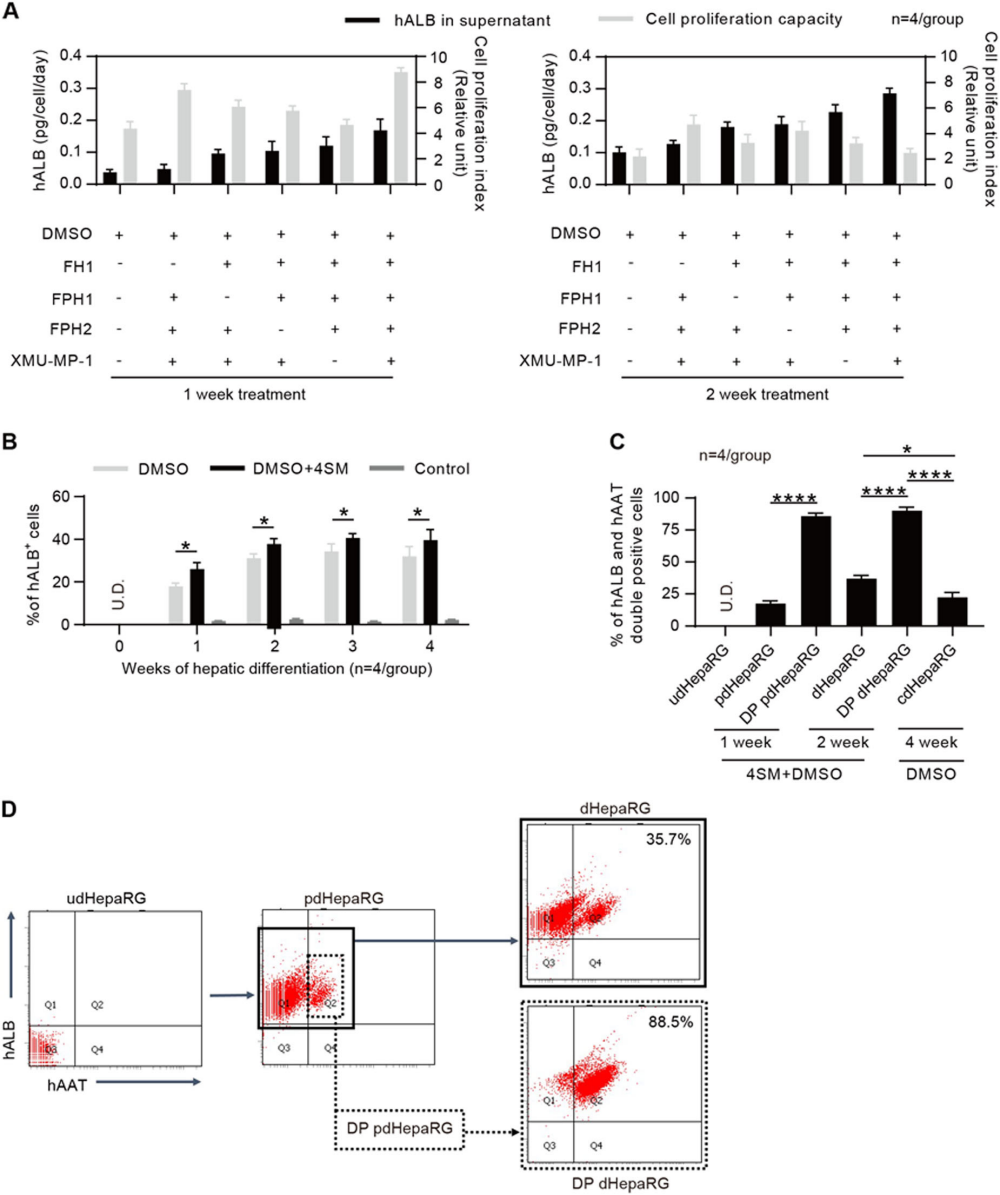
Optimization of hepatic differentiation of HepaRG cells by 4SM treatment and cell enrichment. **a** Measurements of hALB in the supernatant of the culture medium and cell proliferation capacity of HepaRG cells with the FH1, FPH1, FPH2 and XMU-MP-1 treatment in the one-minus treatment pattern (*n* = 4/group) for 1 (left panel) or 2 weeks (right panel). **b** FACS analysis for the hALB-positive rate of HepaRG cells treated or untreated with 4SM from weeks 0 to 4 in the hepatic differentiation procedure (*n* = 4/group). **c** FACS analysis of the hALB and hAAT double-positive (DP) rate of cells using the hepatic differentiation procedure that was optimized by 4SM treatment and cell enrichment; udHepaRG and cdHepaRG cells were used as controls. **d** Representative FACS plot for developing DP cells using the 4SM-enhanced hepatic differentiation procedure with or without cell enrichment. (*P < 0.01; ****P < 0.00001; NS no significant difference, U.D. undetectable)

A classical 4-week procedure has been used to induce hepatic differentiation of HepaRG cells with DMSO when HepaRG cells are seeded at a low confluence (approximately 20%). The HepaRG cells in the procedure undergo three stages: proliferation, stationary stage and hepatic differentiation^20^. We have previously found that the functional small molecule FH1 promotes the differentiation of iPSC to HLC in vitro, and the functional small molecule SMU-MP-1 promotes in vitro expansion of engrafted HLCs in vivo^31^. Consequently, we wondered whether 4SM could enhance the differentiation of HepaRG cells. To examine this question, HepaRG cells at 50% confluence were treated with DMSO or different combinations of 4SM (5 μM for each SM) in a one-minus pattern for 1 or 2 weeks (Fig. 1a). The 4SM-treated HepaRG cells showed significantly higher levels of hALB (a mature human hepatocyte marker) in the supernatant than those treated with different 3SM, as shown in Figs. 1a, b. The enhancing effects of 4SM on HepaRG differentiation and proliferation were also observed using the classical 4-week hepatic differentiation procedure (cdHepaRG) (Fig. 1c and Supplementary Fig. 1A, B). Notably, the prehepatic differentiated HepaRG (pdHepaRG) cells treated with 4SM for 1 week (Fig. 1a, left panel) showed higher in vitro cell proliferation capacity than those treated for 2 weeks (Fig. 1a, right panel). Flow cytometry assays showed that 4SM treatment mildly but significantly increased the ratio of hALB-positive (hALB^+^) cells to approximately 40% within 2 weeks, whereas cdHepaRG exhibited approximately 30% without 4SM treatment (Fig. 1b). Our results also showed that a 2-week extension of 4SM treatment did not increase the ratio of hALB^+^ cells (Fig. 1b). These results indicated that 4SM treatment was adequate for inducing mature hepatic differentiation within 2 weeks.

To increase the ratio of hALB^+^ cells in the hepatic differentiation procedure, the precursor HLCs were enriched by collagenase IV treatment of pdHepaRG cells as previously described^30^. Briefly, pdHepaRG cells generated by treating undifferentiated HepaRG (udHepaRG) cells with 4SM + DMSO for a week were treated for a short duration with collagenase IV. CLCs were detached from the dish, suspended in the supernatant and washed off while the precursor HLCs were still attached to the bottom. We performed fluorescence activated cell sorting (FACS) analysis to examine the hepatic markers hALB and hAAT. As shown in Fig. 1c, only 17.5 ± 2.1% of pdHepaRG cells (not treated with collagenase IV) were positive for both hALB and hAAT, and the DP pdHepaRG cells (enriched with collagenase IV) exhibited a DP rate of 85.9 ± 2.3% for hALB and hAAT (Fig. 1c). Therefore, collagenase IV treatment could greatly enrich for the DP cells. The enriched HLCs were then treated with 4SM + DMSO for another week, resulting in more hepatic differentiation of DP pdHepaRG cells, named DP dHepaRG cells, which reached 90.2 ± 2.9% of the DP rate for hALB and hAAT (Fig. 1c). Without enrichment using collagenase IV, the udHepaRG cells, HLCs derived from pdHepaRG (dHepaRG) cells with only 4SM + DMSO treatment, and cdHepaRG cells treated with only DMSO showed a DP rate at 0%, 37.1 ± 2.5%, and 22.4 ± 3.8%, respectively (Fig. 1c). A representative plot from the FACS assay to show the DP marker (hAAT and hALB) for pdHepaRG, dHepaRG, DP pdHepaRG and DP dHepaRG cells in the hepatic differentiation procedure is shown in Fig. 1d.

**Fig. 2:**
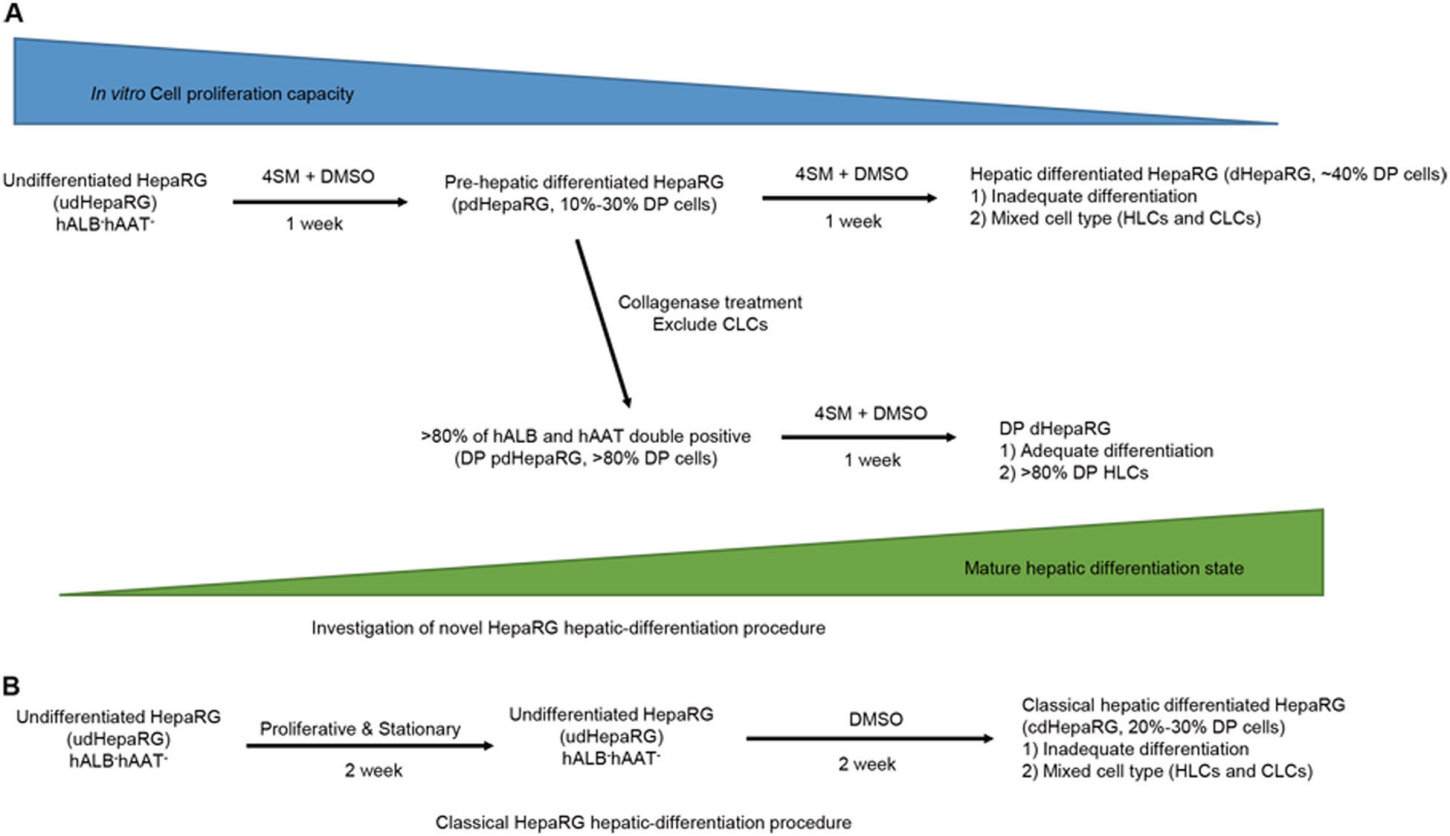
**Schematic diagram of the hepatic differentiation procedure for HepaRG cells**. **a** Investigation of the optimized 2-week hepatic differentiation procedure including 4SM treatment and enrichment of precursor HLCs (DP pdHepaRG). **b** Classic 4-week hepatic differentiation procedure with only DMSO treatment

Interestingly, although DP dHepaRG cells displayed higher hALB secretion, DP pdHepaRG cells displayed a similar proliferation capacity to pdHepaRG cells and a much higher proliferation rate than DP dHepaRG or dHepaRG cells (Supplementary Fig. 1A, B). These results suggested that enrichment of DP pdHepaRG cells significantly increased the ratio of cells that were in a relatively mature and hepatic differentiation state by eliminating most of the nonprecursor HLCs. Thus, we developed a novel 2-week hepatic differentiation procedure for HepaRG cells by 4SM + DMSO treatment and cell enrichment (Fig. 2a), which is advantageous for the classical 4-week hepatic differentiation procedure (Fig. 2b) in terms of saving time and achieving a higher differentiation rate.

### Characterization of cdHepaRG, dHepaRG and DP dHepaRG cells

**Fig. 3:**
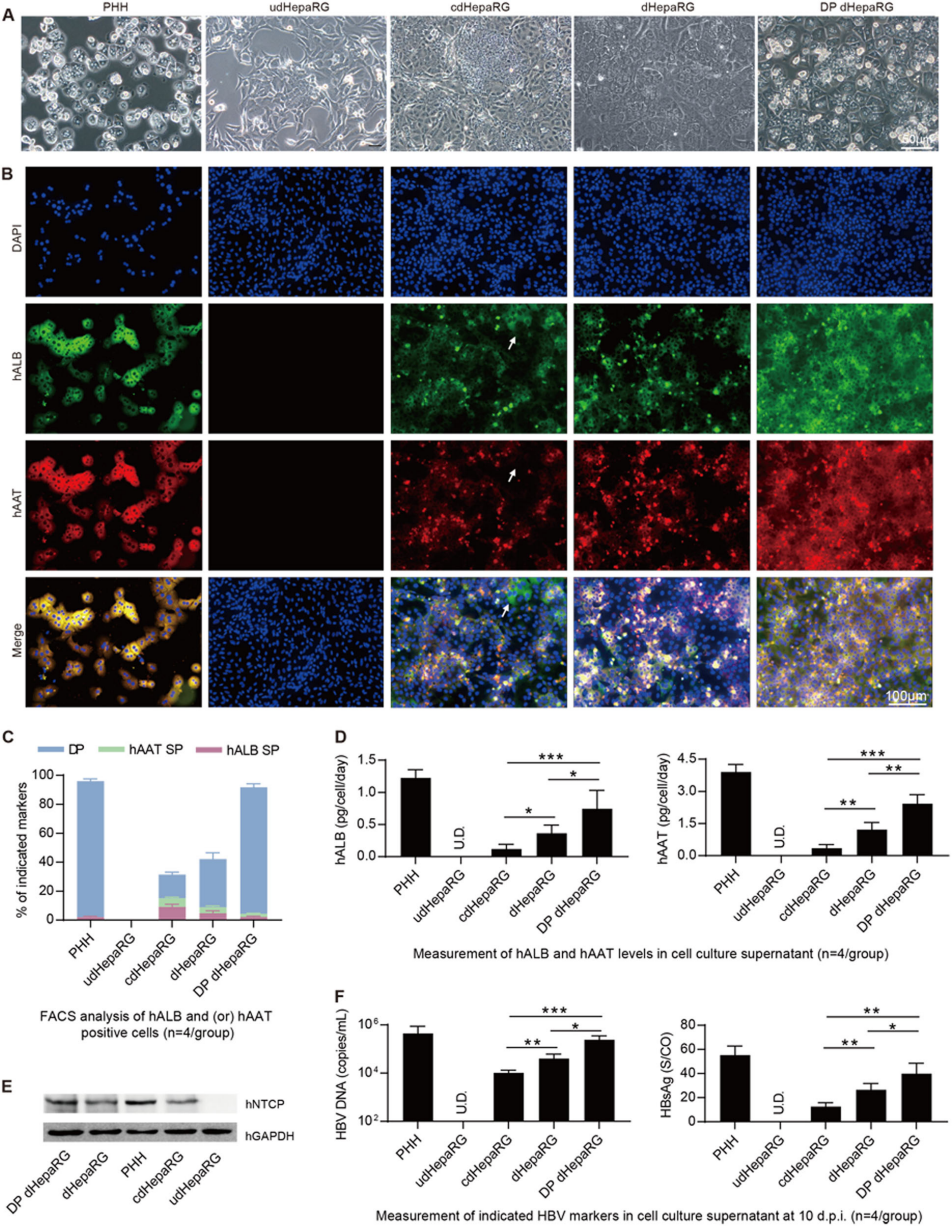
**Characterization of cdHepaRG, dHepaRG and DP dHepaRG cells***.* **a** Morphology of PHH, udHepaRG, cdHepaRG, dHepaRG and DP dHepaRG cells (bar = 50 μm). **b** IF staining for hALB (green), hAAT (red) and nuclei (blue) of PHH, udHepaRG, cdHepaRG, dHepaRG and DP dHepaRG cells. The single-positive cells in cdHepaRG are indicated by white arrowheads. PHH was used as a positive control (bar = 100 μm). **c** FACS analysis of either hALB- or hAAT- or double-positive PHH, udHepaRG, cdHepaRG, dHepaRG and DP dHepaRG cells (*n* = 4/group). **d** ELISA measurements of hALB (left panel) or hAAT (right panel) levels in the cell culture supernatant of PHH, udHepaRG, cdHepaRG, dHepaRG and DP dHepaRG cells (*n* = 4/group). **e** Western blot examination of the expression of control (hGAPDH) or hNTCP protein in PHH and HepaRG cells in different hepatic differentiation states. **f** Measurements of HBV DNA (left panel) or HBsAg (right panel) levels in the cell culture supernatant of PHH or HepaRG cells in different hepatic differentiation states at 10 days post-HBV infection (*n* = 4/group). (*P < 0.01; **P < 0.001; ***P < 0.0001; NS no significant difference, U.D. undetectable)

After obtaining HepaRG cells with hepatic differentiation, their susceptibility to HBV was characterized. First, we wondered whether the morphology differed among the different cell lines. Thus, we observed the cells (PHH, udHepaRG, cdHepaRG, dHepaRG and DP dHepaRG) under a microscope and obtained images as shown in Fig. 3a. In contrast to PHH, udHepaRG cells showed fibroblast-like morphology. The other cell lines shared different rates of similarities in morphology to PHH: approximately 30% for cdHepaRG cells, approximately 50% for dHepaRG cells, and >90% for DP dHepaRG cells (Fig. 3a). Next, we employed an immunofluorescence (IF) method to examine hAAT and hALB in cdHepaRG, dHepaRG and DP dHepaRG cells. PHH and udHepaRG cells were used as the positive and negative control, respectively. As shown in Fig. 3b, >90% of DP dHepaRG cells and PHHs were DP, whereas <50% of dHepaRG cells were DP. Approximately 40% of dHepaRG cells were DP, and only ∼20% of cdHepaRG cells were DP and some hALB positive (indicated by the arrowheads in Fig. 3b). Next, we performed FACS analysis to count the DP or single-positive cell numbers and obtained results consistent with those of IF with more single-positive (either hAAT or hALB) cells observed in cdHepaRG cells (Fig. 3c). We were then curious whether the cells could secrete hAAT and hALB into the cell culture medium, and thus, we examined the level of hAAT and hALB in the cell supernatant. We found that DP dHepaRG cells secreted less hALB and hAAT than PHH but more than other cells (Fig. 3d). Importantly, DP dHepaRG cells expressed higher levels of HBV receptor, sodium taurocholate co-transporting polypeptide (hNTCP) than dHepaRG or cdHepaRG cells, which indicated a mature hepatocyte phenotype (Fig. 3e and Supplementary Fig. 1C). Moreover, quantitative reverse transcriptase polymerase chain reaction (qRT-PCR) results showed that DP dHepaRG cells expressed more mRNAs of hepatic-specific genes than dHepaRG or cdHepaRG cells (Supplementary Fig. 1d). Finally, we wanted to examine the permissiveness of the cells to HBV infection. Thus, we infected the cells with HBV (genotype D) at a multiplicity of infection (MOI) of 200 copies/cell for 10 days and examined the HBV DNA (Fig. 3f, left) and HBsAg (Fig. 3f, right). DP dHepaRG cells yielded >10-fold more HBV DNA than the cdHepaRG cells and significantly higher amounts of HBV DNA than dHepaRG cells. Therefore, DP dHepaRG cells were more permissive for in vitro HBV infection than dHepaRG and cdHepaRG cells but less permissive than PHH (Fig. 3f). Taken together, the in vitro-cultured DP dHepaRG cells were more hepatic differentiated, with a hepatic phenotype closer to PHHs, and hence were more susceptible to HBV infection than dHepaRG or cdHepaRG cell.

### Generation of HepaRG-FRGS mice by engraftment of DP pdHepaRG cells and 4SM treatment

**Fig. 4:**
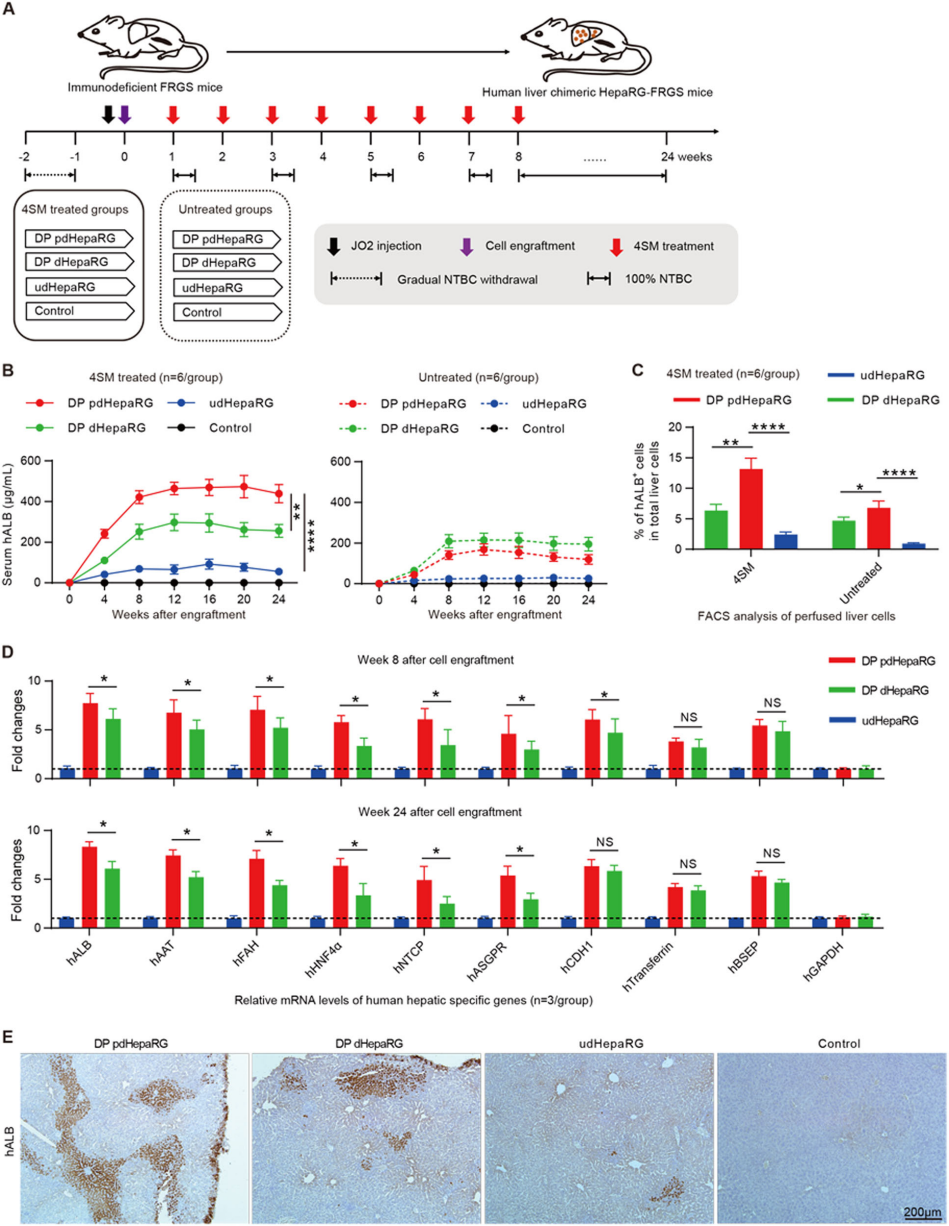
**Generation and characterization of HepaRG-FRGS mice**. **a** Schematic diagram of the experimental plan for engraftment of HepaRG cells in different hepatic differentiation states to liver failure FRGS mice with or without 4SM treatment. FRGS mice without engraftment were used as a control. **b** ELISA assays to determine the serum hALB levels at weeks 0 to 24 post-engraftment (*n* = 6/group). **c** FACS analysis to examine the ratio of hALB^+^ cells in the liver of the engrafted FRGS mice with 4SM treatment at weeks 8 and 24 after engraftment. FRGS mice without cell engraftment were used as a control (*n* = 6/group). **d** qRT-PCR to detect the mRNA levels of control (hGAPDH) or nine typical human hepatocyte-specific genes in hALB^+^ cells from the engrafted FRGS mice treated with 4SM at week 8 and 24 after engraftment (*n* = 3/group). **e** IHC to visualize hALB-positive cells in liver tissues from the engrafted FRGS mice with 4SM treatment at week 24 post-engraftment (bar = 200 μm). (*P < 0.01; **P < 0.001; ****P < 0.00001; NS no significant difference, U.D. undetectable)

Previous studies have demonstrated that both the efficiency of liver chimerism and the degree of hepatic differentiation are important for the chimeric liver to support long-term HBV infection^16,27^. We wondered whether HepaRG cells could be used to develop a chimeric liver in mouse for HBV infection. For this purpose, we engrafted the DP dHepaRG cells with mature hepatic differentiation (but low in vitro proliferation capacity) and DP pdHepaRG cells with middle mature hepatic differentiation (but high in vitro proliferation capacity) to FRGS mice. The udHepaRG-engrafted FRGS mice and the mice without cell engraftment were used as controls. To investigate the in vivo effect of 4SM on HepaRG differentiation, the mice were divided into two groups: one group was treated weekly with 4SM from weeks 1 to 8 after engraftment, and the other group was not treated (Fig. 4a). Prior to engraftment, liver injury of FRGS mice was induced by 2-[2-nitro-4-(trifluoromethyl) benzoyl] cyclohexane-1,3-dione (NTBC) cycling and the mouse CD95 antibody JO2 to kill a portion of the mouse liver cells to provide space for the expansion of implanted cells. To maintain stable liver chimerism, NTBC was administered to the mice in drinking water from weeks 8 to 24 after engraftment (Fig. 4a). First, we performed enzyme-linked immunosorbent assay (ELISA) assays to examine the serum hALB levels of all engrafted mice, and as shown in Fig. 4b, the hALB levels increased from weeks 0 to 8 and were maintained at peak levels until 24 weeks post-engraftment. The hALB levels in the 4SM-treated mice (left panel) were higher than those in the untreated mice with the same cell engraftment (right panel). The mice engrafted with DP pdHepaRG, DP dHepaRG or udHepaRG cells and with 4SM treatment exhibited serum hALB levels of 421.6 ± 78.3 μg/mL, 251.8 ± 89.9 μg/mL and 68.9 ± 23.8 μg/mL, respectively, at week 8 post-engraftment, which were maintained until week 24 (Fig. 4b, left). The mice engrafted with DP pdHepaRG, DP dHepaRG or udHepaRG cells and without 4SM treatment exhibited serum hALB levels of 140.8 ± 21.8 μg/mL, 209.2 ± 24.1 μg/mL and 24.3 ± 2.8 μg/mL, respectively, at week 8 post-engraftment, which were maintained until week 24 (Fig. 4b, right). Total liver cells of the engrafted mice treated with 4SM were collected by liver collagenase perfusion for further analysis. A FACS analysis demonstrated that the rate of hALB^+^ cells in total liver of DP pdHepaRG, DP dHepaRG or udHepaRG-engrafted mice with 4SM treatment reached 11.9 ± 3.7%, 6.5 ± 2.1% and 2.3 ± 0.8%, respectively, at week 24 post-engraftment (Fig. 4c, left). However, the rate of hALB^+^ cells in total liver of DP pdHepaRG, DP dHepaRG or udHepaRG-engrafted mice without 4SM treatment reached 6.8 ± 1.9%, 4.7 ± 1.1% and 0.9 ± 0.2%, respectively, at week 24 post-engraftment (Fig. 4c, right). The serum hALB levels and the rate of hALB^+^ cells in liver indicated a linear correlation among the different groups of engrafted mice, which were considered methods of choice to estimate liver chimerism (Supplementary Fig. 2A).

Next, we set out to evaluate the expression of hepatocyte-specific genes. The hALB^+^ cells from 4SM-treated mice were collected to isolate total RNA at week 8 or 24 post-engraftment. qRT-PCR was carried out to measure mRNA levels of the hepatocyte-specific genes, as indicated in Fig. 4d. Interestingly, the hALB^+^ cells from DP pdHepaRG-engrafted mice displayed slightly but significantly higher levels of human hepatic-specific genes than DP dHepaRG and much higher levels of all human hepatocyte-specific genes than udHepaRG-engrafted mice at week 8 or week 24 post-engraftment (Fig. 4d), which indicated that DP pdHepaRG cells exhibited relatively mature hepatic differentiation. However, DP dHepaRG cells displayed more hepatic differentiation than DP dHepaRG or udHepaRG cells in vitro (see Fig. 3). Moreover, a western blot assay showed higher hNTCP expression in hALB^+^ cells from DP pdHepaRG-engrafted mice than from DP dHepaRG-engrafted mice (Supplementary Fig. 2B, C). Interestingly, FACS analysis and IF assay demonstrated that hNTCP and hALB were presented simultaneously in both DP pdHepaRG- and DP dHepaRG-engrafted mice (Supplementary Fig. 2D, E). Finally, an immunohistochemistry (IHC) assay was performed to examine hALB from liver tissues from the 4SM treatment groups at week 24 post-engraftment. As shown in Fig. 4e, the chimeric liver of DP pdHepaRG-engrafted mice exhibited higher liver chimerism than that of DP dHepaRG- or udHepaRG-engrafted mice (Fig. 4e). Taken together, these results showed that DP pdHepaRG cells had a better capacity for both proliferation and hepatic differentiation potential in vivo and represented an ideal cell source for engraftment in FRGS mice. Consequently, we used the DP pdHepaRG-engrafted FRGS mice with 4SM treatment, termed HepaRG-FRGS, for subsequent HBV infection and therapy studies.

### Establishment of chronic HBV infection in HepaRG-FRGS mice

**Fig. 5:**
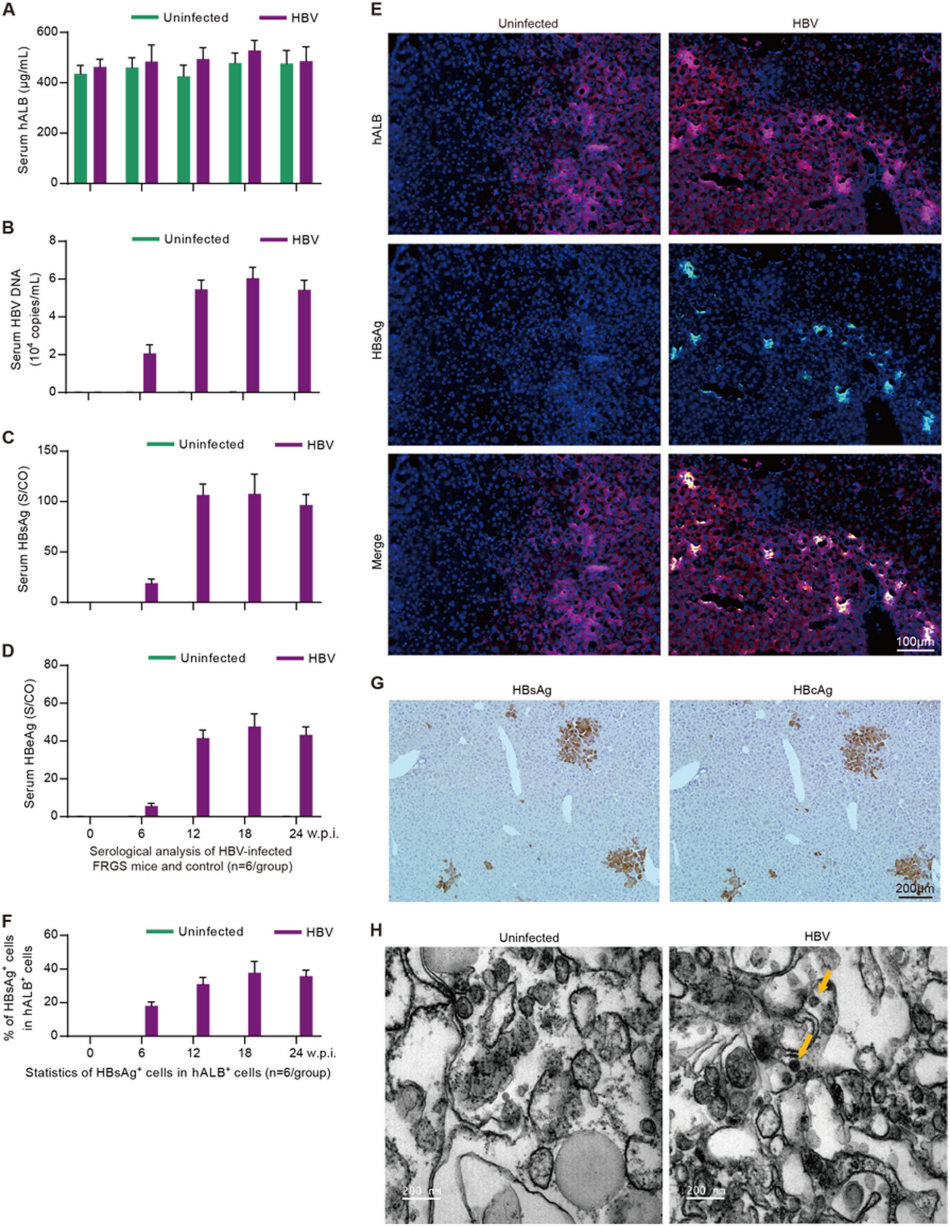
**Establishment of chronic HBV infection in HepaRG-FRGS mice***.* ELISA to determine the levels of serum (**a**) hALB, (**c**) HBsAg and **d** HBeAg, and qRT-PCR to determine the levels of **b** HBV DNA in uninfected control and HBV-infected HepaRG-FRGS mice from 0 to 24 w.p.i. (*n* = 6/group). **e** IF assay of hALB and HBsAg-positive cells in frozen section collected from liver tissues of the uninfected control and HBV-infected HepaRG-FRGS mice at 12 w.p.i. (bar = 100 μm). Cell nuclei were stained with DAPI. **f** Statistical analysis of the ratio of HBsAg^+^ cells in hALB^+^ cells in different views (*n* = 6/group). **g** IHC assay of HBsAg and HBcAg expression in serial sections collected from HBV-infected HepaRG-FRGS mice at 12 w.p.i. (bar = 200 μm). **h** ITEM images of intracellular HBV virions in hALB-positive cells collected from the uninfected control and HBV-infected HepaRG-FRGS mice. The HBV virions were labeled with 5-nm colloidal gold particles (bar = 200 nm)

Next, we wanted to determine whether the HepaRG-FRGS mice could be infected by HBV. Thus, HepaRG-FRGS mice were intraperitoneally inoculated with 1 × 10^6^ DNA copies of purified HBV. The serum hALB levels of the uninfected control or HBV-infected HepaRG-FRGS mice were maintained at approximately 500 μg/mL throughout the 24-week infection course (Fig. 5a). IHC staining of HBV-infected HepaRG-FRGS liver revealed a considerable number of cells that were positive for hNTCP from 0 to 24 weeks post-infection (w.p.i.), which was critical for HBV infection (Supplementary Fig. 3A). Moreover, we also detected productive amplification of HBV in HepaRG-FRGS over a 24-week course: (1) serum HBV DNA levels increased to 10^4^–10^5^ copies/mL (Figs. 5b), (2) serum HBsAg levels increased to 100–150 S/CO and (3) HBeAg increased to 40–60 S/CO at week 12 (Figs. 5c, d). The levels of HBV production were maintained up to 24 w.p.i.. In contrast, these HBV infection markers were undetectable in the uninfected HepaRG-FRGS mice with similar serum hALB levels (Figs. 5a, d, green column). These serological results confirmed the establishment of chronic HBV viremia in HBV-infected HepaRG-FRGS. To visualize HBV infection in the chimeric liver, we performed IHC after collecting the liver tissues from the HBV-infected HepaRG-FRGS mice at 12 w.p.i. to detect the human liver proteins hALB (red) and HBsAg (green). As shown in Fig. 5e, HBsAg was detected in the hALB-positive cells. After counting the hALB-positive and hALB/HBsAg duel-positive cells, we found that 30–40% of the hALB-positive cells in liver lobes were positive for HBsAg; this ratio was maintained until 24 w.p.i. (Fig. 5f). IHC staining of serial sections showed that >90% of the HBsAg-positive cells were also positive for HBcAg at 12 w.p.i. (Fig. 5g). The immune transmission electron microscopy (ITEM) assay results showed the morphology of the 40–50 nm diameter HBV virions in hALB^+^ cells from HBV-infected HepaRG-FRGS mice (Fig. 5h).

**Fig. 6:**
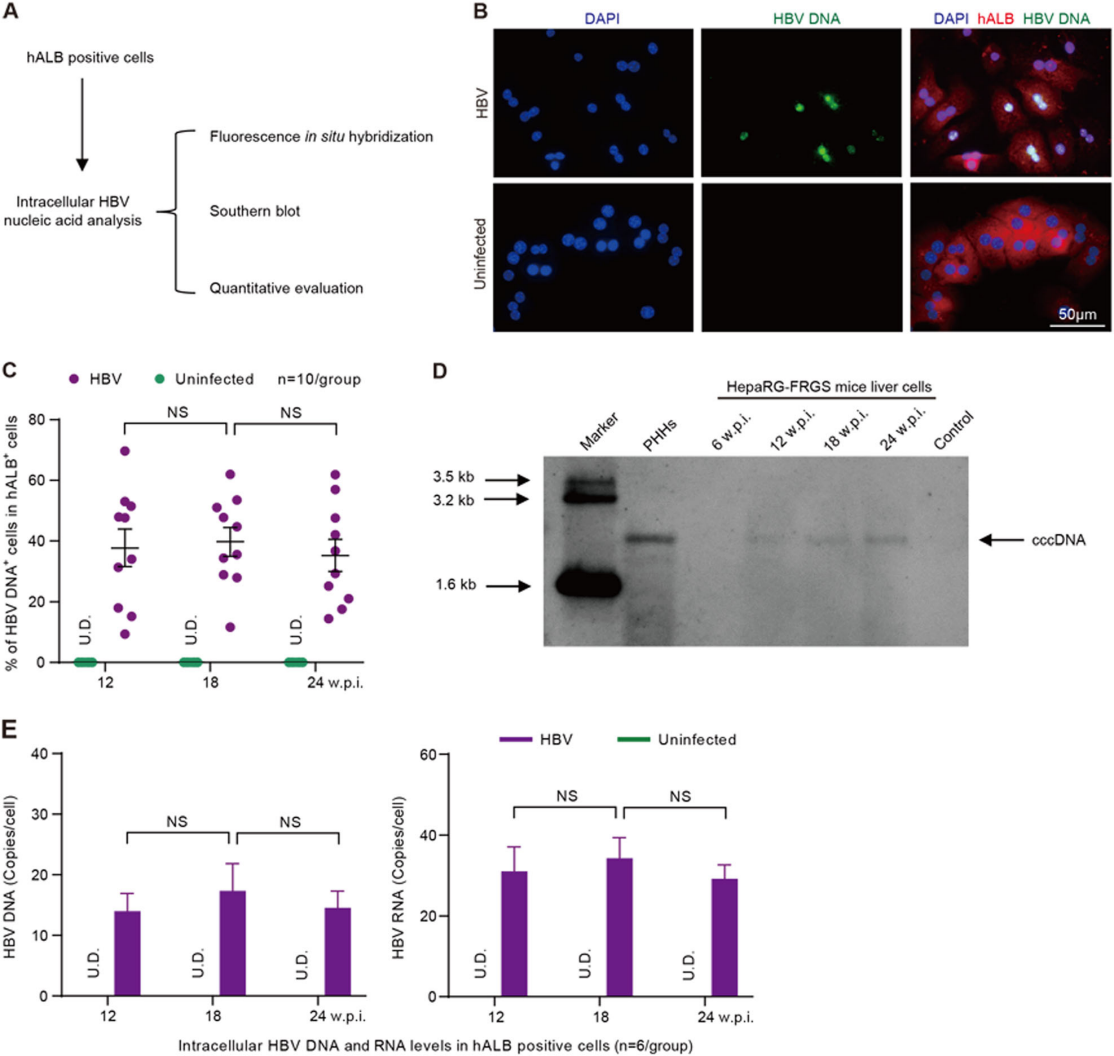
**Analysis of intracellular HBV DNA and RNA.** **a** Schematic diagram of intracellular HBV DNA and RNA in hALB-positive cells from the liver of HBV-infected HepaRG-FRGS mice. **b** FISH analysis to visualize HBV DNA replication and determine the ratio of HBV DNA^+^ cells (green) in hALB^+^ cells (red) from the uninfected control or HBV-infected HepaRG-FRGS mice at 12 w.p.i. (bar = 50 μm). **c** Statistics for the ratio of HBV DNA^+^ cells in different views of the FISH analysis (*n* = 10/group). **d** Southern blot analysis to detect intracellular HBV cccDNA in hALB^+^ cells from the uninfected control (line 7) or HBV-infected HepaRG-FRGS from 6 to 24 w.p.i. (lines 3–6) and from HBV-infected PHHs (line 2). **e** Measurement of intracellular HBV DNA and RNA levels in hALB-positive cells collected from uninfected control and HBV-infected HepaRG-FRGS mice from 12 to 24 w.p.i. (*n* = 6/group). (NS no significant difference, U.D. undetectable)

To investigate intrahepatic HBV infection markers, hALB^+^ cells were collected from HBV-infected HepaRG-FRGS mice and were used for more biological analysis (Fig. 6a). First, fluorescence in situ hybridization (FISH) analysis was carried out to detect replicated HBV DNA. Our results showed that HBV DNA replication occurred in 37.8 ± 16.5% of hALB^+^ cells at 12 w.p.i., and the positive rate was maintained until 24 w.p.i. (Figs. 6 b, c). Importantly, Southern blot assays confirmed that the infected cells synthesized intracellular HBV cccDNA in hALB^+^ cells from HBV-infected HepaRG-FRGS mice from 12 to 24 w.p.i. (Fig. 6d). As shown in Fig. 5e, the intracellular HBV RNA and HBV DNA levels in these hALB^+^ cells were 15.5 ± 6.1 copies/cell and 7.1 ± 2.9 copies/cell at 12 w.p.i., respectively, and were maintained at these levels until 24 w.p.i. (Fig. 6e). Additionally, HepaRG-FRGS mice were also capable of supporting chronic infection of HBV genotype A, B or C (Supplementary Fig. 3b to 3E). As expected, the udHepaRG or DP dHepaRG cells engrafted to FRGS with 4SM treatment displayed lower serum hALB levels and lower or undetectable viral loads during a similar 24-week HBV (genotype D) infection course (Supplementary Fig. 4A to 4D). These results showed that HepaRG-FRGS mice had considerable liver chimerism, and a mature hepatic differentiation state of implanted cells was adequate to support the complete life cycle of HBV infection and establish chronic viremia.

### Evaluation of novel antivirals that block viral spreading in HBV-infected HepaRG-FRGS mice

**Fig. 7:**
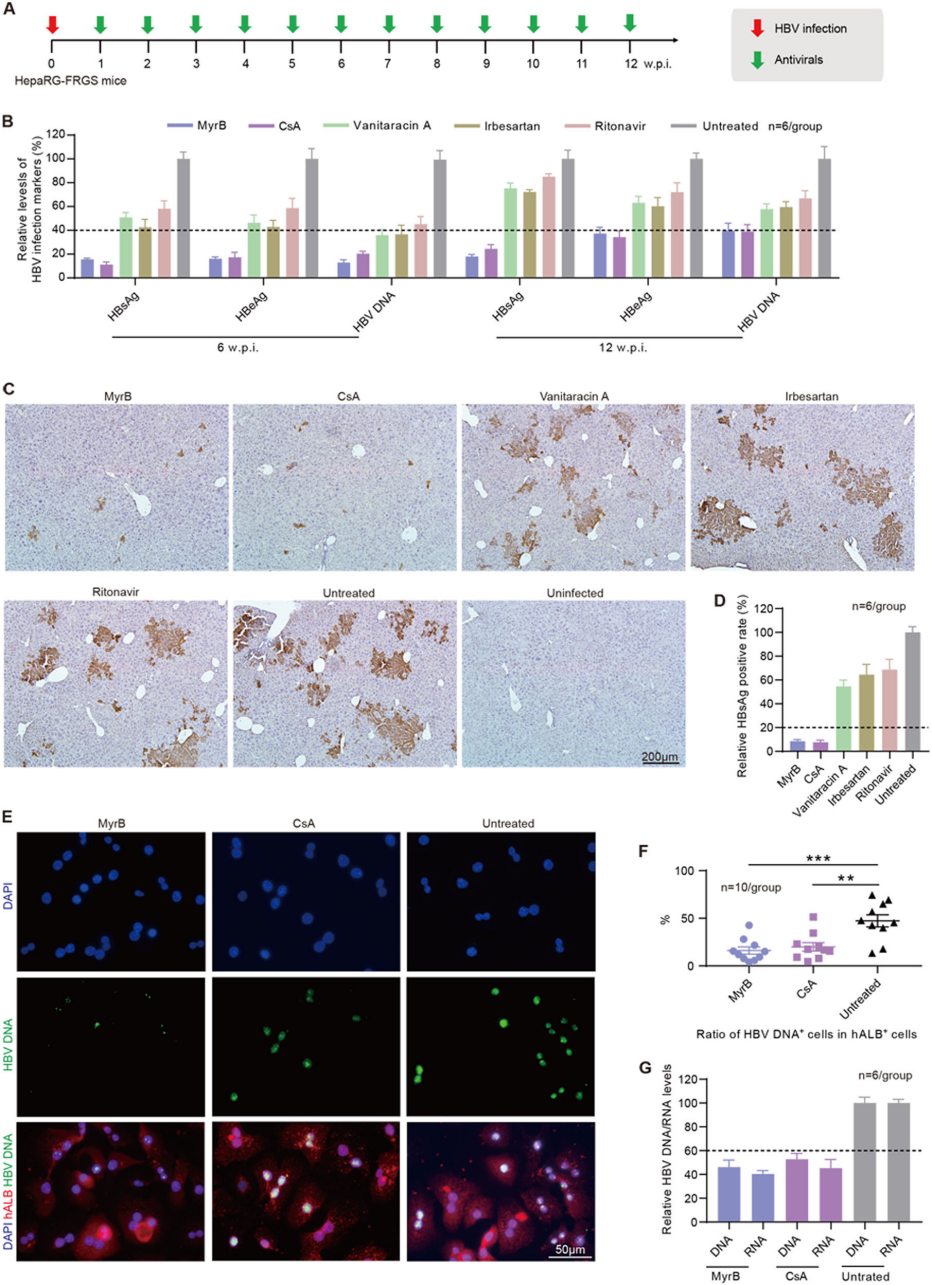
**Evaluation of anti-HBV drugs that block viral spreading in HBV-infected HepaRG-FRGS mice**. **a** Schematic diagram of the treatment of anti-HBV entry inhibitors in HBV-infected HepaRG-FRGS mice. **b** Serum HBsAg, HBeAg and HBV DNA levels of the untreated control and HBV-infected FRGS mice with anti-HBV entry inhibitor treatment at 6 and 12 w.p.i. (*n* = 6/group). **c** IHC staining for HBsAg^+^ cells in liver tissues collected from untreated control and HBV-infected FRGS mice with anti-HBV entry inhibitor treatment at 12 w.p.i. (bar = 200 μm). **d** Statistics for HBsAg^+^ cells in liver tissues in different views (*n* = 6/group). **e** FISH analysis of HBV DNA^+^ cells in hALB^+^ cells collected from untreated control and HBV-infected FRGS mice with MyrB or CsA treatment at 12 w.p.i. (bar = 50 μm). **f** Statistics for HBV DNA^+^ cells in hALB^+^ cells in different views (*n* = 10/group). **g** Measurement of intracellular HBV DNA and RNA levels in hALB^+^ cells collected from untreated control and HBV-infected FRGS mice with MyrB or CsA treatment at 12 w.p.i. (*n* = 6/group). (**P < 0.001; ***P < 0.0001; NS no significant difference, U.D. undetectable)

The HTAs that block HBV entry were effective in preventing viral spreading^32^. Here, we set out to evaluate the antiviral effects of several HBV entry inhibitors, including myrcludex B (MyrB), cyclosporin A (CsA), vanitaracin A, irbesartan and ritonavir^33–37^. HBV-infected HepaRG-FRGS mice were treated weekly with each of the entry inhibitors from 1 to 12 w.p.i. (Fig. 7a). First, we observed that treatment with the drugs did not affect the serum hALB levels of the HepaRG-FRGS mice, which were all maintained at approximately 500 μg/mL from 0 to 12 w.p.i., indicating stable liver chimerism (Supplementary Fig. 5A). Second, we found that MyrB significantly suppressed HBV production, as shown in Fig. 7b, by 81.9 ± 3.2% for HBsAg, 62.5 ± 6.3% for HBeAg, and 60.4 ± 4.9% for HBV DNA at 12 w.p.i. CsA played a similar suppressive effect on HBV infection. In contrast, the other three drugs (vanitaracin A, irbesartan and ritonavir) showed only 20–40% suppression of HBsAg, HBeAg and HBV DNA in serum (Fig. 7b). In comparison with the untreated HBV-infected HepaRG-FRGS mice, the IHC results showed that MyrB or CsA resulted in a 91.5 ± 3.5% or 92.3 ± 4.2% decrease in HBsAg^+^ cells in liver tissues from HepaRG-FRGS at 12 w.p.i. (Figs. 7 c, d). The other three drugs showed only a 20–40% decrease in HBsAg^+^ cells at the same time point (Figs. 7 c, d). Additionally, hALB^+^ cells were collected from the MyrB and CsA treatment groups for further analysis of intracellular HBV infection markers. FISH analysis of HBV DNA-positive cells showed that MyrB and CsA treatment significantly suppressed the ratio of HBV DNA^+^ cells in collected hALB^+^ cells (Figs. 7 e, f). In comparison with the untreated mice, MyrB treatment showed 52.8 ± 6.7% suppression of intracellular HBV DNA and 59.4 ± 2.1% suppression of intracellular HBV RNA levels at 12 w.p.i. (Fig. 7g). CsA treatment showed 47.2 ± 5.3% suppression of intracellular HBV DNA and 54.7 ± 7.2% suppression of intracellular HBV RNA levels at the same time point (Fig. 7g).

It is important to identify whether antiviral drugs have any side effects on mice. Thus, we performed different assays. First, we examined the hALB levels and the body weights of the mice used for the drug experiment. We found that hALB levels and body weights were not significantly altered by the drugs (Supplemental Fig. 5a, b). Liver function assays were performed to examine the levels of alanine transaminase (ALT), aspartate aminotransferase (AST), total bilirubin (TBIL) and total bile acid (TBA), and no significant variations were detected in the mice treated with the drugs compared with those without drugs (Supplemental Fig. 5c). Therefore, our HepaRG-FRGS mice can be used to evaluate anti-HBV drugs, and MyrB and CsA are the most effective drugs for suppressing HBV replication.

## Discussion

PHH and iPSC-HLC have been used to generate chimeric livers in mice, and the developed humanized mouse model has been useful to investigate pathogenesis of, antiviral effects on, and virological characteristics of HBV and hepatitis C virus (HCV)^17,18^. However, both PHH and iPSC-HLC are expensive, and PHH has limited availability and poor expandability^38^. Thus, an alternative cell type is needed, which must be well differentiated hepatically so that it can be permissive for HBV infection and able to expand in vitro and in vivo to generate human liver chimeric mice with adequate liver chimerism for HBV infection. The hepatoma-derived cell lines support HBV replication and particle assembly following the transfection of cloned viral genomes, but they fail to complete the complete viral replication cycle, especially for receptor-mediated virion entry and cccDNA formation^4,5,39^. The hNTCP-overexpressing hepatoma cell lines also have limited applications because (1) their transformed mode makes them differ from PHHs in many physiological characteristics, (2) they have lost several host factors that are important for HBV replication and (3) they lack normal innate immune responses to HBV^27^. HLCs derived from stem cells (hiPSCs or hESCs) have been demonstrated to be promising novel tools to study HBV and HCV infection, and they have been used to generate human liver chimeric mice for in vivo HBV infection study until our recent successful attempt^38,40^. The only drawback of using iPSCs is that the induction procedure for stem cells is expensive and complex.

Previous studies from different groups have demonstrated that HLCs derived from HepaRG cells might be an important alternative for PHH because HepaRG-derived HLCs presented hepatocyte phenotypes, including a hepatocyte-like morphology and the expression of hALB, hAAT, hNTCP and cytochrome P450, and more importantly, they have been used for in vitro HBV infection and for studying innate immune response and antiviral therapy^24,41–44^. However, a mixed cell phenotype (half HLCs and half CLCs) exists in the cultured HepaRG cells and causes a low HBV infection efficiency, hindering their further development for a human liver chimeric mouse model for HBV infection^18^. In this study, we used collagenase IV treatment to eliminate CLCs and enrich for precursor HLCs derived from HepaRG cells, resulting in DP pdHepaRG cells. The procedure was then optimized using 4SM to increase the ratio of cells with relatively mature hepatic differentiation up to approximately 90%. Therefore, we solved the issue of a mixed cell phenotype during the hepatic differentiation of HepaRG cells. Indeed, mature hepatic differentiation of the host cells is important to support HBV infection^27^. The results showing more efficient in vitro HBV infection in DP dHepaRG cells than in cdHepaRG and dHepaRG cells confirmed this theory. Additionally, the effect of 4SM treatment promoted hepatic differentiation and cell proliferation, suggesting the potential of small molecules in the modulation of cell fate and state. In comparison with the classical 4-week hepatic differentiation procedure for HepaRG cells, we developed a new hepatic differentiation procedure that reduced the induction time to 2 weeks and increased the hepatic differentiation rate and cell proliferation capacity, resulting in a significantly high ratio of HLCs.

Several groups have previously attempted to generate humanized liver mice using HepaRG cells or HepaRG-derived HLCs. In their studies, udHepaRG cells or HepaRG-derived HLCs (induced only with DMSO, called cdHepaRG) were engrafted to mouse liver. Their results showed low liver chimerism of the mice^26,45,46^. In addition, all the mice had low serum hALB levels (<50 μg/mL) and only a few HLCs distributed in the liver lobes. CLCs were also observed in liver lobes of the udHepaRG-engrafted mice and were not permissive for HBV infection. In the present study, the DP pdHepaRG-engrafted FRGS mice with 4SM treatment showed a much higher hALB level (>400 μg/mL), approximately 10% liver chimerism, and a high positive rate of hNTCP in hALB-positive cells at week 8 after engraftment. More importantly, the levels of hepatocyte-specific proteins were maintained for at least 16 weeks. Another important finding in the present study was that 4SM exerted better effects on the implanted DP pdHepaRG to cause higher cellular proliferation and more hepatic differentiation than on the implanted DP dHepaRG cells after 8 weeks of in vivo treatment (Figs. 4b, e), which suggested that 4SM was more effective on the precursor HLCs than the HLCs. Taken together, the improved proliferation capacity and hepatic differentiation are two critical conditions in establishing a HepaRG-engrafted mouse model for HBV infection.

HBV infection is mostly chronic and persists for a long time in patients. Therefore, a human liver chimeric mouse model must not only maintain mature hepatic differentiation but also support HBV infection in vivo for a long term^16–19^. The HepaRG-FRGS mice developed in this study displayed considerable chimerism and hepatic differentiation of implanted HLCs, and they supported chronic HBV infection. Sustained production of HBV DNA, HBsAg and HBeAg suggested chronic HBV viremia during the infection course. The detected intracellular HBV DNA and RNA indicated viral replication and transcription. The HBsAg and HBcAg expressed in liver lobes displayed productive infection. Notably, the sustained existence of HBV cccDNA in liver confirmed the establishment of chronic HBV infection. The severity of HBV-caused hepatitis and the large population of HBV infection urged us to develop anti-HBV drugs. However, in recent decades, the lack of infectious animal models has been one of the main obstacles hindering the development and evaluation of novel antivirals^32^. To date, as many novel antivirals have been developed and evaluated in preclinical studies for other viruses, an infectious animal model would be a useful tool for a comprehensive understanding of their mechanism and in vivo evaluation of their anti-HBV effects. In this study, we evaluated the therapeutic effect of several HBV entry inhibitors on HBV infection and replication in HepaRG-FRGS mice. The suppressive effects of the anti-HBV drugs were detected, and the results are consistent with those reported by other groups^32^. These experimental results indicated that the HepaRG-FRGS mice were applicable not only to support chronic HBV infection but also to evaluate the use of novel antivirals against HBV infection. Although the optimized HepaRG cells (DP pdHepaRG) in this study could overcome the lack of PHHs in the generation of the humanized liver chimeric mouse to some extent, HepaRG-FRGS still presented some drawbacks. For example, the HepaRG-FRGS mice generated a lower load of HBV than the PHH-engrafted mice, mainly due to a relatively lower chimerism and hepatic differentiation. Therefore, improving the proliferation capacity and the hepatic maturity of the optimized HepaRG cells will be the main directions of our future studies.

In conclusion, our creative use of 4SM and collagenase IV to treat HepaRG cells helped us to develop a novel hepatic differentiation procedure and to overcome the disadvantages of the classical hepatic differentiation procedure. The established HepaRG-FRGS mice provide a novel approach to generate humanized liver chimeric mice with an available and convenient cell source, which may become a useful infectious animal model for HBV infection and therapy studies, as well as other hepatotropic virus infections and liver diseases.

## Materials and methods

### HepaRG culture and passage

udHepaRG cells were cultured in 10-cm plates in basic William’s E medium (WME, A12176-01, GIBCO) supplemented with 10% fetal bovine serum (FBS; 10099-141, GIBCO), 100 units/mL penicillin-streptomycin (V900929, Sigma-Aldrich), 5 μg/mL insulin (I9278, Sigma-Aldrich) and 5 × 10^−5^ M hydrocortisone (HC, CC-4335BB, LONZA), and passaged every 2 weeks (1/5 dilution) by trypsinization. Cells were cultured at 37 °C with 5% CO_2_.

### Classical 4-week hepatic differentiation procedure for HepaRG cells

As previously described, cells were initially seeded at a low confluence (approximately 20%) and cultured in basic WME for 2 weeks. They were then maintained in the differentiation WME: basic WME supplemented with 2% DMSO (D2650, Sigma-Aldrich) for 2 more weeks. The medium was renewed every 2 or 3 days^20^. Cells were cultured at 37 °C with 5% CO_2_. After the classical 4-week hepatic differentiation procedure, we obtained the classical differentiated HepaRG (cdHepaRG) with mixed phenotypes of HLCs and CLCs.

### Optimized 2-week hepatic differentiation procedure for HepaRG cells

The 4SM was purchased from APExBIO, USA (FH1, B3700; FPH1, B3701; FPH2, B4753; XMU-MP-1, A8735). The cells were first cultured to 50% confluence using an optimized differentiation medium (WME supplemented with 1% DMSO, 5 μM FH1, 5 μM FPH1, 5 μM FPH2, 5 μM XMU-MP-1) for 1 week to obtain pdHepaRG. The cells were then cultured at 37 °C with 5% CO_2_. The DP pdHepaRG cells were collected using a cell enrichment procedure and then reseeded at 50% confluence and incubated for another week in the optimized differentiation medium. Thus, we obtained DP dHepaRG cells with mature hepatic differentiation and an approximately 90% DP rate of hALB and hAAT. In contrast, we obtained hepatic differentiated HepaRG (dHepaRG) cells with mixed phenotypes of HLCs and CLCs after 2 weeks of 4SM and DMSO treatment without the cell enrichment procedure.

### Enrichment for hALB and hAAT DP cells

The enrichment procedure was carried out as previously described^30^. To enrich for the hALB and hAAT DP cells, pdHepaRG cells were treated with 2 mg/mL collagenase IV (A004186-0001, Sigma-Aldrich) for 5 min. CLCs were detached from the dish and suspended in supernatant, whereas HLCs were still attached to the bottom. We removed the collagenase IV and washed off the fibroblast-like cells twice with phosphate-buffered saline (PBS). The remaining cells (DP pdHepaRG) were digested with trypsin for engraftment or further hepatic differentiation. The enriched cells were analyzed by FACS and showed that >80% of the cells were DP for hALB and hAAT.

### Ethics statement

All animal experiments were carried out in strict compliance with the Animal Welfare Act, PHS Policy and the standards of the American Association for the Accreditation of Laboratory Animal Care and other national statutes and regulations relating to animals. The animal use protocol was approved by the Institutional Animal Care and Use Committee (IACUC) and Laboratory Animal Management Ethics Committee at Xiamen University (Protocol Number: XMULAC20160049).

### Animal study

To obtain *Fah*^*‒/‒*^*Rag2*^*‒/‒*^*IL-2Rγc*^*‒/‒*^*SCID* (FRGS) mice, the *Fah*^*‒/‒*^*Rag2*^*‒/‒*^*IL-2Rγc*^*‒/‒*^ (FRG) mice described in our previous studies^29,31,47^ were crossed with *BALB/c SCID* mice (Shanghai SLAC Laboratory Animal Co., Ltd, China) in a specific pathogen-free laboratory in the Animal Centre of Xiamen University. The FRGS mice were raised under the protection of NTBC dissolved in the daily drinking water during the breeding period.

### Generation and characterization of HepaRG-FRGS mice

FRGS mice were anesthetized with isoflurane and received splenic injections of 3 × 10^6^ udHepaRG, DP pdHepaRG or DP dHepaRG cells as previously described^8,48^. Liver injury was induced by NTBC (SML0269, Sigma-Aldrich) cycling^48^ and mouse CD95 antibody JO2 (554254, BD Bioscience)^13^ to kill a portion of the mouse liver cells and provide space for the implanted cells, as previously described. NTBC was gradually reduced in the drinking water from weeks −2 to −1 and provided for short periods of time (3 days each time) at weeks 1, 3, 5 and 7 post-engraftment. FRGS mice received 0.2 mg/kg intraperitoneal injections of JO2 at day −1 post-engraftment. To enhance the in vivo expansion of the implanted cells, the mice received weekly intraperitoneal injections of 4SM (1 μg/g for each small molecule) dissolved in 0.1% citric acid aqueous solution containing 20% Kolliphor HS 15 (42966, Sigma-Aldrich). To maintain relatively stable liver chimerism, HepaRG-FRGS mice received 100% NTBC in drinking water without other treatments from weeks 8 to 24 post-engraftment. As previously described, the serum hALB level was used as indicator of liver chimerism^8^. For further analysis, liver cells were collected by two-step liver perfusion of collagenase IV, as previously described^4^. The perfused liver cells were further analyzed and collected by FACS. For IHC staining, liver tissues were collected by partial hepatectomy.

### HBV infection in vitro and in vivo

Genotype A, B, C or D of HBV (isolated and purified previously in our laboratory) was propagated and amplified in HBV-replicating stable cell lines (HepG2-HBV1.3-A, ‒B, ‒C or ‒D) generated from HepG2 cells (ATCC, cat # HB −8065) in our previous studies^49^ and purified as previously described^24^. In brief, the infectious inoculum was prepared from freshly collected supernatants by precipitating viral particles in the presence of 6% PEG. The pellet was resuspended in PBS containing 25% FBS and stored at −80 °C. For in vitro HBV infection, the cells were incubated with an infectious source at an MOI of 200 genome equivalents per cell in culture medium supplemented with 4% PEG 8000 for 20 h at 37 °C. At the end of the incubation, the cells were washed three times with culture medium. During the course of in vitro HBV infection, the medium was changed every 2 days. For the in vivo HBV infection, HepaRG-FRGS mice with approximately 500 mg/mL serum hALB levels were inoculated by intraperitoneal injections of 1 × 10^6^ DNA copies of an infectious source dissolved in normal saline. During the HBV infection course, HepaRG-FRGS mice received 100% NTBC in drinking water, and the samples were collected at the indicated time points.

### Measurement of HBV infection markers

Methods for virological detection of HBV-infected HepaRG-FRGS mice have been detailed described in our previous studies^47,50^. HBsAg and HBeAg in the cell culture supernatant and mouse sera were detected using commercial ELISA kits (Wantai, Beijing, China). Intracellular HBV antigen, DNA and virions were detected by immunohistochemical staining, FISH analysis and immuno transmission electron microscopy, respectively (details are provided in Supplementary Materials and Methods). HBV RNA, DNA and cccDNA were isolated and quantified as previously described^50,51^. The intracellular HBV cccDNA was isolated by the “Hirt” method as previously described^52,53^. Southern blot analysis was performed according to our previous study using DIG-labeled DNA fragments from the X gene as a probe^50^.

### Statistics

Results of the measurements are presented as the mean ± SD. For comparisons between two groups; the unpaired Welch’s *t*-test was applied to calculate the statistical probability in this study (*P < 0.01; **P < 0.001; ***P < 0.0001; ****P < 0.00001; NS no significant difference, U.D. undetectable). For comparisons of more than two groups, one-way analysis of variance followed by Tukey’s post hoc test was applied. Statistical analysis was performed using the GraphPad Prism 7 software.

Further details of the materials and methods, including the instruments, reagents, antibodies (Supplementary Tab. 1) and primers (Supplementary Tab. 2), are provided in the Supplementary Materials and Methods.

## Electronic supplementary material

Supplementary Figure 1

Supplementary Figure 2

Supplementary Figure 3

Supplementary Figure 4

Supplementary Figure 5

Supplementary materials
