# Supplementary figures and images for: Optimized HepaRG is a suitable cell source to generate the human liver chimeric mouse model for the chronic hepatitis B virus infection
Source: Emerg Microbes Infect. 2018 Aug 10;7:144. doi: 10.1038/s41426-018-0143-9 (PMC6086841; doi:10.1038/s41426-018-0143-9)

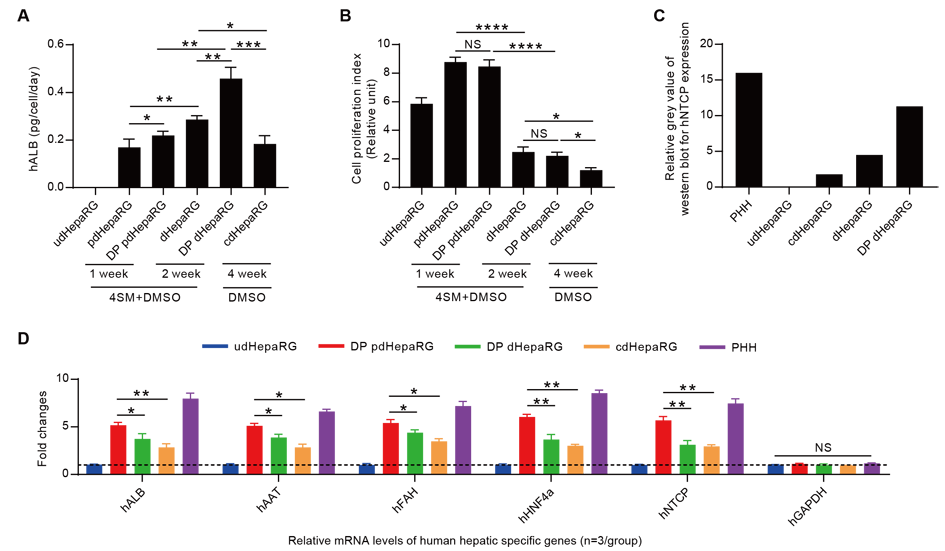

Supplement: Supplementary file 1 — Supplementary Figure 1 [file 41426_2018_143_MOESM1_ESM.tif]

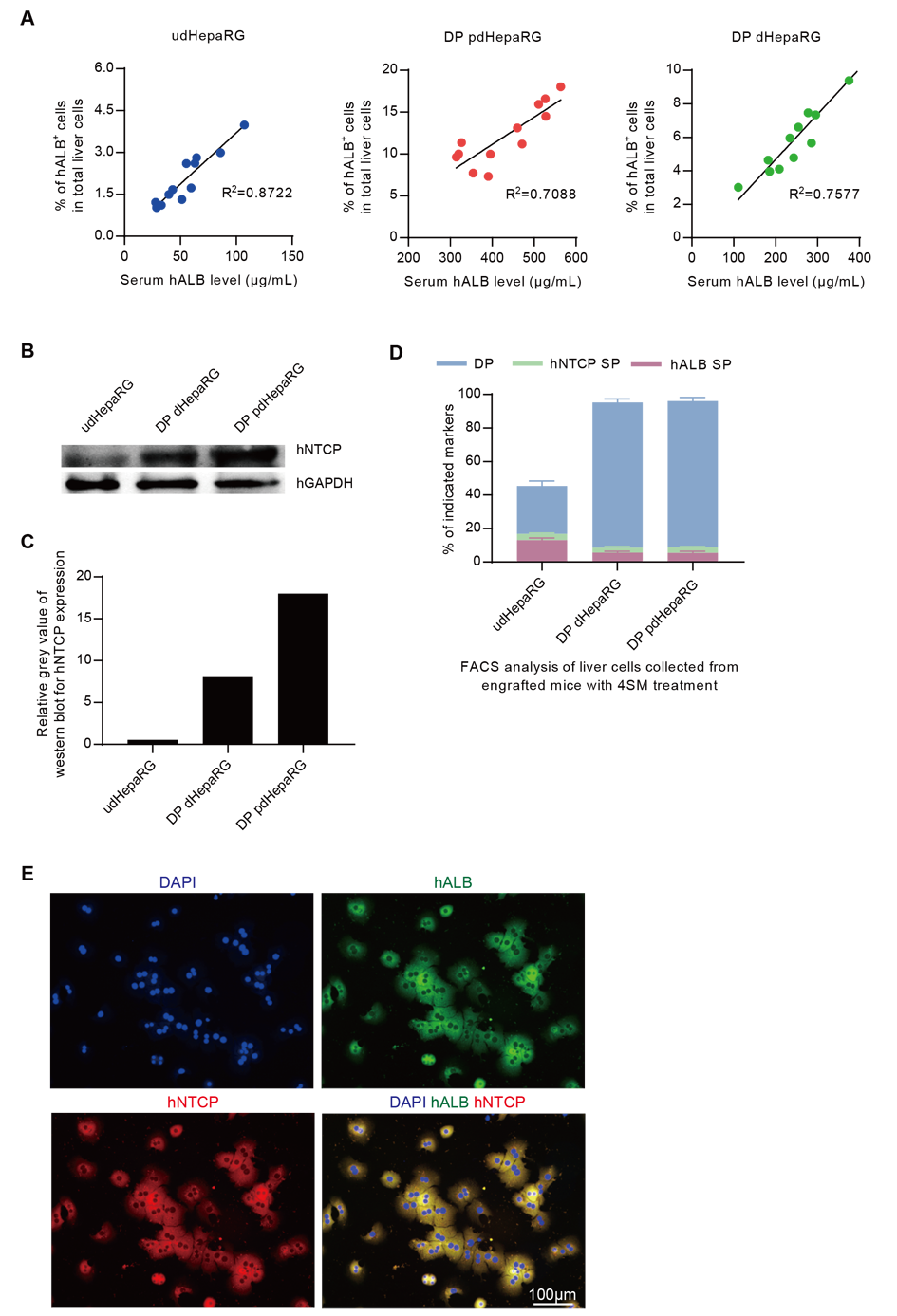

Supplement: Supplementary file 2 — Supplementary Figure 2 [file 41426_2018_143_MOESM2_ESM.tif]

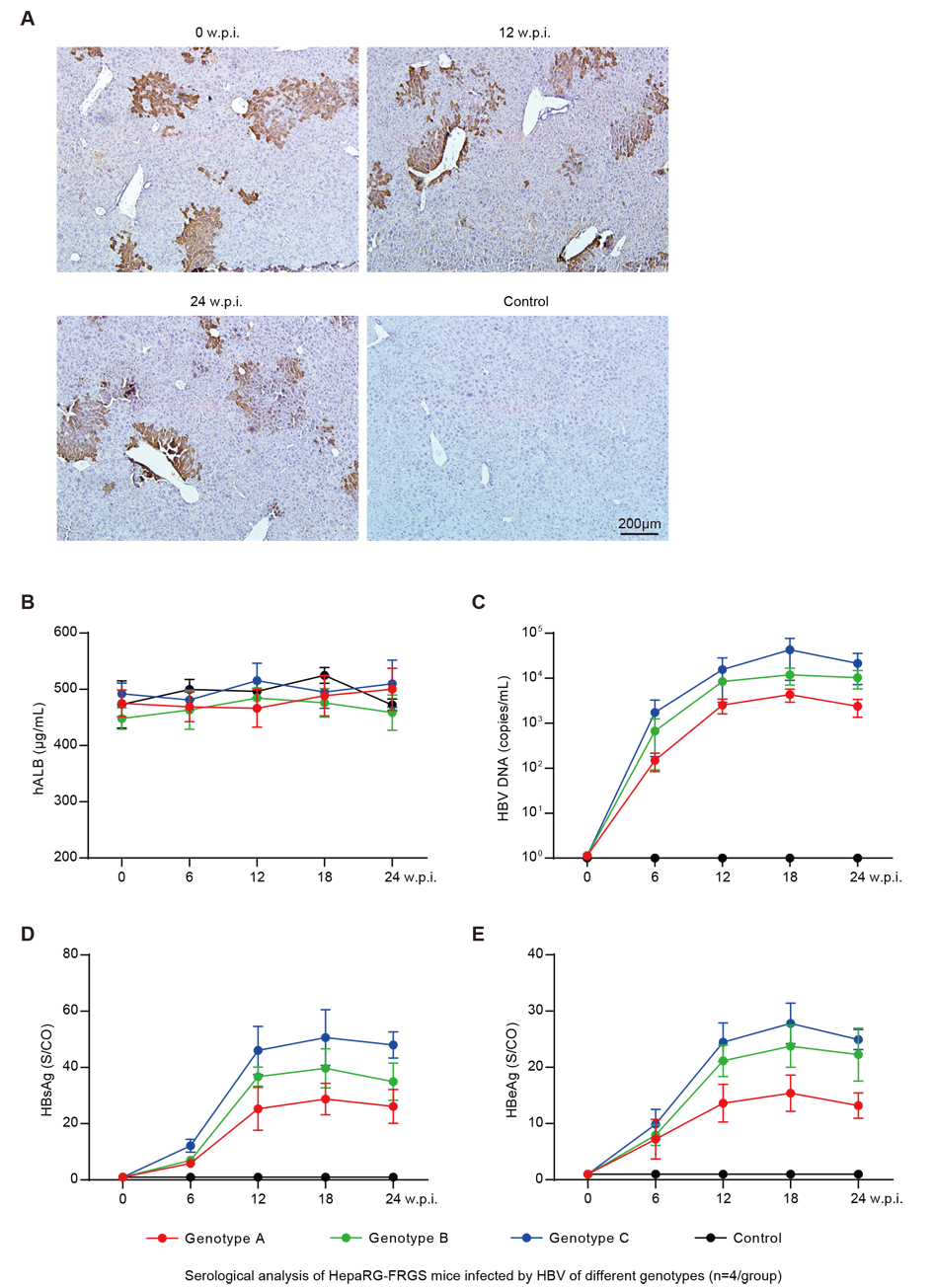

Supplement: Supplementary file 3 — Supplementary Figure 3 [file 41426_2018_143_MOESM3_ESM.tif]

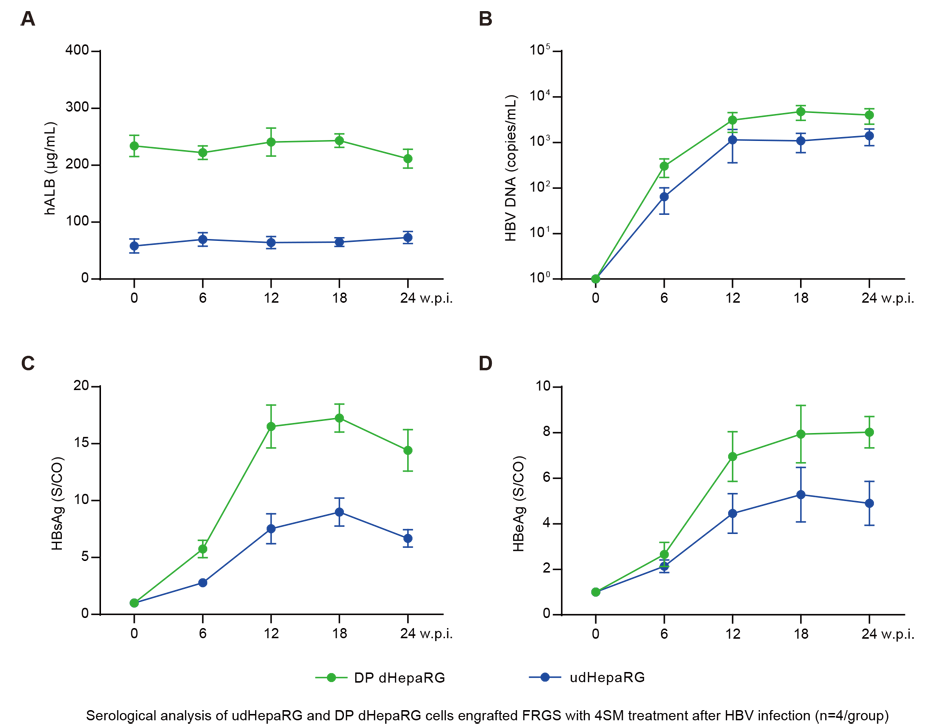

Supplement: Supplementary file 4 — Supplementary Figure 4 [file 41426_2018_143_MOESM4_ESM.tif]

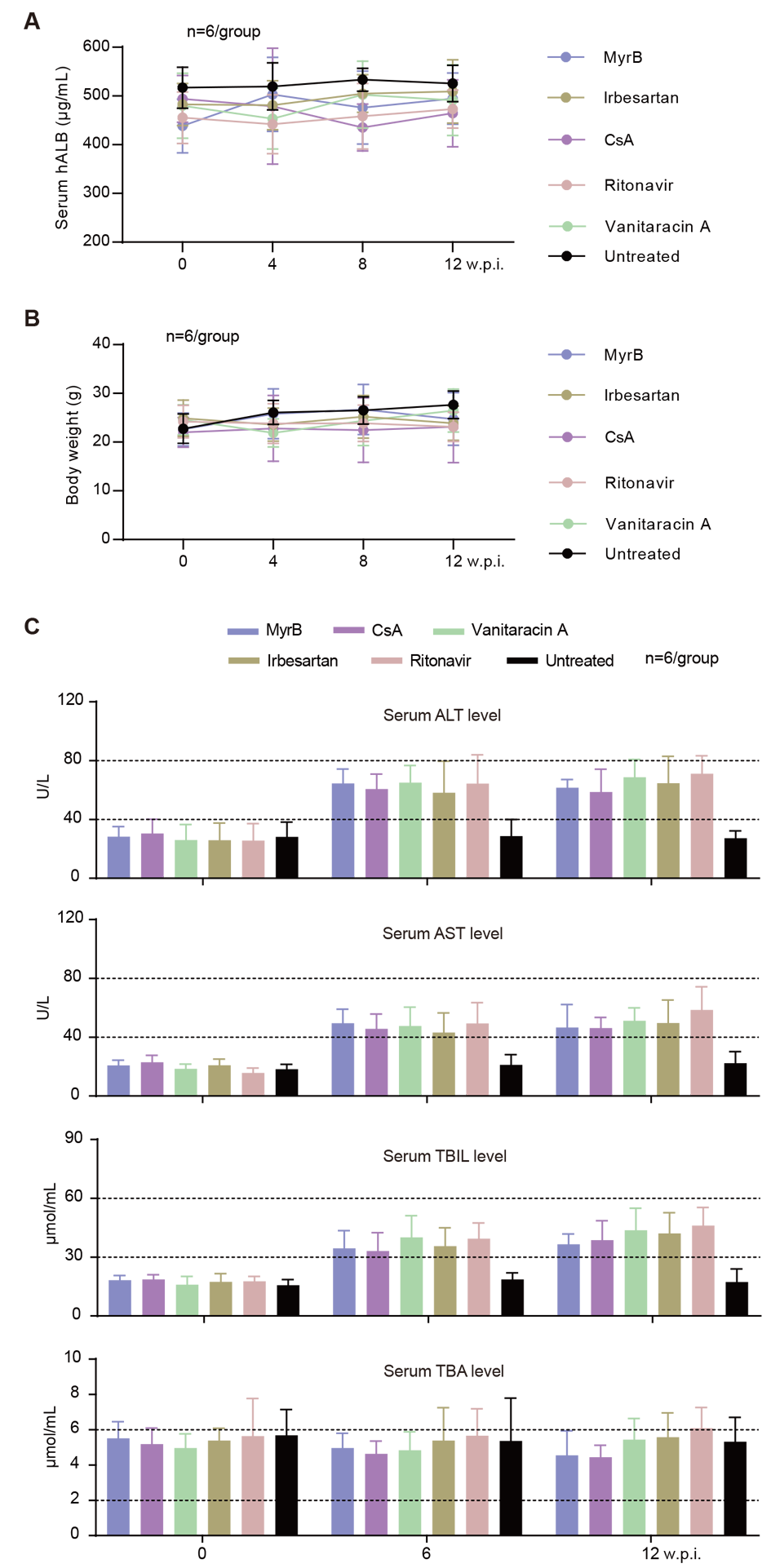

Supplement: Supplementary file 5 — Supplementary Figure 5 [file 41426_2018_143_MOESM5_ESM.tif]
