# Supplementary material for: Optimized HepaRG is a suitable cell source to generate the human liver chimeric mouse model for the chronic hepatitis B virus infection
Source: Emerg Microbes Infect. 2018 Aug 10;7:144. doi: 10.1038/s41426-018-0143-9 (PMC6086841; doi:10.1038/s41426-018-0143-9)
Supplement: Supplementary file 6 — Supplementary materials [file 41426_2018_143_MOESM6_ESM.docx]

**Supplementary Materials**

**Optimized HepaRG is a suitable cell source to generate the human liver chimeric mouse model for the chronic hepatitis B virus infection**

Lunzhi Yuan^1¶^, Xuan Liu^1¶^, Liang Zhang^1¶^, Yali Zhang^1^, Yao chen^1^, Xiaoling Li^1^, Kun Wu^1^, Jiali Cao^1^, Wangheng Hou^1^, Yuqiong Que^1^, Jun Zhang^1^, Hua Zhu^2^, Quan Yuan^1^*, Qiyi Tang^3^*,Tong Cheng^1^*, Ningshao Xia^1^

^1^ State Key Laboratory of Molecular Vaccinology and Molecular Diagnostics, National Institute of Diagnostics and Vaccine Development in Infectious Diseases, School of Life Science, School of Public Health, Xiamen University, Xiamen, 361102, PR China

^2^ Department of Microbiology and Molecular Genetics, New Jersey Medical School, Rutgers University, 225 Warren Street, Newark, NJ 070101, USA

^3^ Department of Microbiology, Howard University College of Medicine, Washington, DC 20059, USA

**Contents**

● **Supplementary Materials and Methods**

○ Detection of cell proliferation ability

○ Quantitation of hALB and hAAT level

○ qRT-PCR

○ FACS analysis

○ Immunofluorescence staining

○ Immunohistochemistry staining

○ Fluorescence *in situ* hybridization analysis

○ Immuno-transmission-electron-microscopy

○ HBV entry inhibitors treatment

○ Western blot

○ Measurement of liver functional markers

**● Supplementary Figure Legends**

**● Reference for Supplementary Materials**

**● Supplementary Tables**

○ Supplementary Tab.1. Antibodies used in this study.

○ Supplementary Tab.2. The qRT-PCR primers used in this study.

**● Supplementary Figures**

**Supplementary Materials and Methods**

***Detection of cell proliferation ability***

1×10^2^ cells were cultured in E-Plates and set in iCELLigence (ACEA Bioscience, San Diego, California, USA) for real-time monitoring of cell proliferation ability. The cell proliferation ability was calculated by the change of cell attachment areas on the bottom of E-Plates between the time point of initial cell attachment and indicated time point.

***Quantitation of hALB and hAAT level***

The level of hALB and hAAT in mice serum or in cell culture supernatant was measured using ELISA Quantitation Kits according to the manufacturer’s protocol (E80-129, E88-122, Bethyl Laboratories, Inc., Montgomery, Canada).

***qRT-PCR***

Total RNA was extracted from tissues or purified cells with TRIzol reagent (Invitrogen) according to the manufacturer’s instructions and used for cDNA synthesis with the SuperScript First-Strand Synthesis System (Invitrogen). Quantitative reverse transcription (RT)-PCR was performed on a 7500 Fast Real-Time PCR system.

***FACS analysis***

The cells for FACS analysis were incubated at 4℃ for 30 minutes with indicated antibodies. They were then rinsed with PBS twice and analyzed with a FACS instrument (Facsaria III, BD). The sorted cells were washed with 0.1 M citric acid (washed off the antibody) for 1 minute before the next step such as FISH or detection of intracellular HBV components.

***Immunofluorescence assay***

Cells were cultured on slides in a 6-well plate and were fixed by 4% paraformaldehyde for 20 minutes, treated by 0.1% Triton-X100 for 10 minutes, incubated with 20% BSA for 30 minutes, and then incubated with the first and second antibodies. After each reaction, the slides were washed with PBS for three times.

***Immunohistochemistry assay***

Mice liver tissues were fixed in 4% formaldehyde (PH 7.4) for 48 hours. Sections (4 µm) were applied to poly-L-lysine-coated slides. After the sections were dewaxed, rehydrated and washed, endogenous peroxidases were inactivated with 3% H_2_O_2_ for 10 minutes. The sections were then incubated overnight with primary antibodies. The sections were subsequently washed with PBS for three times and treated with UltraSensitiveTM SP Kit (Maixin Biotech. Co., Ltd., Fuzhou, China) for the rest steps. Brown staining indicated positive expression. The Sections were visualized using an inverted microscope (BX51, Olympus, Japan), and digital images were captured using Olympus Cell Sense software.

***Fluorescence in situ hybridization analysis***

Before Fluorescence in situ hybridization (FISH) analysis, hALB positive cells were collected from perfused mice liver cells and cultured on slides in 6-well plate for 24 hours. HBV DNA was detected by a digoxin (DIG)-labelled HBV X-specific probes. Then, the signal was amplified by a cascade reaction of a biotin-anti-DIG secondary antibody and TRITC-labelled anti-biotin third antibody. Views scanning and analysis were performed with an inverted microscope (AXIO 2.0, Zeiss, Germany) and Metasystem software.

***Immuno-transmission-electron-microscopy***

The steps of immuno-transmission-electron-microscopy (ITEM) were similar to previous described [[1](#_ENREF_1)], 1×10^5^ cells were then fixed for 10 min in PBS containing 4% paraformaldehyde, permeabilized by incubation for 15 min with 0.5% Triton X-100, and incubated for 1 hour at room temperature with PBS containing 1% BSA. They were then incubated overnight at 4°C with mouse monoclonal antibody against HBsAg in PBS containing 1% BSA, washed three times in PBS, and incubated for 1 hour at room temperature with 5 nm colloidal gold-conjugated anti-mouse antibody in PBS. The samples were then washed, postfixed with 1% osmium tetroxide, dehydrated in a graded series of ethanol, and embedded in Epon resin (Sigma). Ultrathin sections (70 nm) were collected on copper grids and stained with 2% uranyl acetate before examination on Tecnai G2 Spirit transmission electron microscope.

***HBV entry inhibitors treatment***

The peptide MyrB was synthesized (by GL Biochem, Ltd., Shanghai, China) as previous described [[2](#_ENREF_2)]. For each treatment, mice received subcutaneously injection with 3 μg/g body weight. Vanitaracin A was synthesized as previous described [[3](#_ENREF_3), [4](#_ENREF_4)]. CsA was immunosuppressant clinically used for suppression of the immunological failure of xenograft tissues (S2286, Selleck Chemicals, USA). Irbesartan and Ritonavir were FDA approved drugs. For each treatment, mice received intraperitoneal injection of 3 μg/g body weight of CsA, or Irbesartan, or Ritonavir separately dissolved in 0.1% citric acid aqueous solution containing 20% Kolliphor HS 15 (Sigma).

***Western blotting***

The western blotting assays were operated as previously described [[5](#_ENREF_5)]. Normalization for gray scale of the western blotting results were operated by the Image J software.

***Measurement of liver functional markers***

Serum ALT, AST, TBIL, TBA level were measured by regents form Wantai, Beijing, China.

**Supplementary Figure Legends**

***Supplementary Fig. 1. Characterization of HepaRG cell in varied differentiation states*.** Measurements for **(A)** hALB in supernatant and **(B)** cell proliferation capacity of *in vitro* cultured HepaRG cell in varied differentiation states (n=4/group). **(C)** Relative grey value of western blot for hNTCP expression levels in *in vitro* cultured HepaRG cell in varied differentiation states, PHH was set as control (Referred to Fig. 3E). **(D)** The mRNA levels of control (hGAPDH) and five typical human hepatic-specific genes in PHHs and *in vitro* cultured HepaRG cell in varied differentiation states were measured by qRT-PCR (n=3/group). (*, P<0.01; **, P<0.001; ***, P<0.0001; ****, P<0.00001; NS, no significant difference; U.D., undetectable).

***Supplementary Fig. 2. Characteristization of the liver cells collected from HepaRG engrafted FRGS mice with 4SM treatment.* (A)** Relationship between serum hALB levels and ratios of hALB^+^ cells in total liver cells FRGS mice engrafted udHepaRG, DP pdHepaRG and DP dHepaRG cells with 4SM treatment (n=12/group). **(B)** Western blot assay for hNTCP expression in hALB^+^ cells collected from udHepaRG (line 1), DP dHepaRG (line 2) and DP pdHepaRG (line 3) engrafted mice with 4SM treatment at 24 weeks after cell engraftment. (**C)** Normalized Relative grey value of western blot results. **(D)** FACS analysis for the ratios of hALB and hNTCP co-positive cells in hALB^+^ cells collected from the udHepaRG, DP pdHepaRG and DP dHepaRG engrafted mice with 4SM treatment at week 24 after engraftment (n=6/group). **(E)** IF staining for hALB and hNTCP expression in hALB^+^ cells collected from DP pdHepaRG engrafted mice with 4SM treatment at week 24 after engraftment (bar=100μm).

***Supplementary Fig. 3. IHC staining for hNTCP expression throughout the 24-week infection course and serological analysis of HepaRG-FRGS mice infected by HBV in different genotypes.* (A)** IHC staining for hNTCP expression in liver tissues collected from HepaRG-FRGS mice infected with HBV (genotype D) from 0 to 24 w.p.i., liver tissue of FRGS mice without cell engraftment or HBV infection was set as control (bar=200μm). HepaRG-FRGS mice were also infected by HBV in genotypes A (red), B (green) and C (blue). Serum **(B)** hALB, **(C)** HBV DNA, **(D)** HBsAg, and **(E)** HBeAg levels of uninfected control and HBV-infected HepaRG-FRGS mice were measured from 0 to 24 w.p.i. (n=4/group).

***Supplementary Fig. 4. Serological analysis of HBV infection outcomes in udHepaRG and DP dHepaRG engrafted mice with 4SM treatment.*** The udHepaRG (blue) and DP dHepaRG (green) engrafted mice with 4SM treatment were infected by HBV (genotype D) at eight weeks after cell engraftment. Serum **(A)** hALB, **(B)** HBV DNA, **(C)** HBsAg, and **(D)** HBeAg levels of uninfected control and HBV-infected HepaRG-FRGS mice were measured from 0 to 24 w.p.i. (n=4/group).

***Supplementary Fig. 5. Liver toxicity analysis for HBV entry inhibitors treatment.***

**(A)** Serum hALB, **(B)** body weight and **(C)** four typical liver function markers of the untreated control and HBV-infected HepaRG-FRGS mice received antiviral treatment from 0 to 12 w.p.i. (n=6/group).

**Reference for Supplementary Materials**

[1] Huang HC, Chen CC, Chang WC, Tao MH, Huang C. Entry of hepatitis B virus into immortalized human primary hepatocytes by clathrin-dependent endocytosis. Journal of virology 2012;86:9443-9453.

[2] Petersen J, Dandri M, Mier W, Lutgehetmann M, Volz T, von Weizsacker F, et al. Prevention of hepatitis B virus infection in vivo by entry inhibitors derived from the large envelope protein. Nature biotechnology 2008;26:335-341.

[3] Kaneko M, Watashi K, Kamisuki S, Matsunaga H, Iwamoto M, Kawai F, et al. A Novel Tricyclic Polyketide, Vanitaracin A, Specifically Inhibits the Entry of Hepatitis B and D Viruses by Targeting Sodium Taurocholate Cotransporting Polypeptide. Journal of virology 2015;89:11945-11953.

[4] Matsunaga H, Kamisuki S, Kaneko M, Yamaguchi Y, Takeuchi T, Watashi K, et al. Isolation and structure of vanitaracin A, a novel anti-hepatitis B virus compound from Talaromyces sp. Bioorganic & medicinal chemistry letters 2015;25:4325-4328.

[5] Zhang TY, Yuan Q, Zhao JH, Zhang YL, Yuan LZ, Lan Y, et al. Prolonged suppression of HBV in mice by a novel antibody that targets a unique epitope on hepatitis B surface antigen. Gut 2016;65:658-671.

**Supplementary Tables**

| ***Supplementary Table 1. Antibodies used in this study*** | | | |
| --- | --- | --- | --- |
| Antibodies | Application | Source | Cat. No. |
| [Purified Anti-Mouse CD95 Clone JO2](http://oa.nidvd.org/material/mPeek.aspx?id=15137) | Induced liver faluire | BD Biosciences | 554254 |
| Anti-HBsAg | IHC; ITEM | Home keeping | 83H12 |
| Anti-human albumin (hALB) | FACS; IF; IHC; FISH |  | 5D2 |
| Goat Anti-Mouse IgG (5nm Gold) preadsorbed | ITEM | Abcam, Cambridge | ab27244 |
| Anti-human α-1 antitryptase (hAAT) | FACS; IF |  | ab9399 |
| Anti-Digoxigenin | FISH |  | ab420 |
| Anti-Biotin antibody (FITC) | FISH |  | ab6650 |
| Anti-Rabbit IgG (whole molecule)–TRITC | second antibody | SIGMA-ALDRICH | T6778-1ML |
| Anti-Mouse IgG (whole molecule)−TRITC |  |  | T5393-1ML |
| Anti-Rabbit IgG (whole molecule)–FITC |  |  | F9887-1ML |
| Anti-Mouse IgG (whole molecule)–FITC |  |  | F9006-2ML |
| Anti-human NTCP | IF |  | HPA042727 |
| Anti-HBcAg | IHC | DAKO | B058601 |

***Supplementary Table 2. The qRT-PCR primers used in this study***

| Gene | NCBI number | Length | Primer | |
| --- | --- | --- | --- | --- |
|  |  |  |  |  |
| ALB | NM_000477.5 | 90 | F | TTTATGCCCCGGAACTCCTTTT |
|  |  |  | R | ACAGGCAGGCAGCTTTATCAG |
| AAT | NM_000295.4 | 233 | F | GCCTATGATGAAGCGTTTAGGC |
|  |  |  | R | TTCCAGTAATGGACAGTTTGGGT |
| HNF4A | NM_000457.4 | 108 | F | AACGGACAGATGTGTGAGTGG |
|  |  |  | R | CAGGAGCTTATAGGGCTCAGAC |
| FAH | NM_000137.2 | 206 | F | CCTACGGCGTCTTCTCGAC |
|  |  |  | R | CTGCAAGAACACTCTCGCCT |
| NTCP | NM_003049.3 | 74 | F | AAGGACAAGGTGCCCTATAAAGG |
|  |  |  | R | ACGATCCCTATGGTGCAAGGA |
| Transferrin | NM_001063.3 | 199 | F | TGTCTACATAGCGGGCAAGTG |
|  |  |  | R | GTTCCAGCCAGCGGTTCTG |
| BSEP | NM_003742.2 | 75 | F | TTGGCTGATGTTTGTGGGAAG |
|  |  |  | R | CCAAAAATGAGTAGCACGCCT |
| CYP3A4 | NM_017460.5 | 174 | F | AAGTCGCCTCGAAGATACACAA |
|  |  |  | R | AAGGAGAGAACACTGCTCGTG |
| ASGPR1 | NM_001671.4 | 241 | F | ATGACCAAGGAGTATCAAGACCTT |
|  |  |  | R | TGAAGTTGCTGAACGTCTCTCT |
| TTR | NM_000371.3 | 188 | F | GGCTCACAACAGATGAGAAA |
|  |  |  | R | TGTGGTGGAGTAAGAGTAGG |
| GAPDH | NM_002046.5 | 242 | F | GGAGTCAACGGATTTGGTCGT |
|  |  |  | R | CACTTGATTTTGGAGGGATCTCG |
